# Comparison of Efficacy between Non-Steroidal Anti-Inflammatory Drugs and Anti-Vascular Endothelial Growth Factor in Preventing Macular Edema after Cataract Surgery in Diabetic Patients

**DOI:** 10.3390/jpm12030351

**Published:** 2022-02-25

**Authors:** Chia-An Hsu, Sheng-Chu Chi, Yu-Bai Chou

**Affiliations:** 1 Department of Ophthalmology, Taipei Veterans General Hospital, Taipei 11217, Taiwan; nfwya0811@gmail.com (C.-A.H.); b101100033@tmu.edu.tw (S.-C.C.); 2 School of Medicine, National Yang Ming Chiao Tung University, Taipei 11217, Taiwan

**Keywords:** anti-inflammatory agents, non-steroidal, anti-VEGF, diabetes mellitus, cataract extraction, macular edema

## Abstract

(1) Background: There is no consensus regarding the optimal strategy to prevent macular edema after cataract surgery in diabetic patients. The purpose of study is to compare the efficacy of topical nonsteroidal anti-inflammatory agents (NSAIDs) and intravitreal injections of anti-VEGFs for the prevention of macular edema after cataract surgery in diabetic patients without pre-existing macular edema. (2) Methods: A literature search of the MEDLINE, PUBMED, and EMBASE databases was conducted in July 2021. Studies involving either topical NSAIDs or intravitreal injections of anti-VEGF arms that reported either the occurrence of macular edema or changes in best corrected visual acuity (BCVA) were included. Weighted mean differences and risk ratios were calculated along with 95% confidence intervals. (3) Results: Intravitreal injection of anti-VEGFs provided short-term structural protection for one month in patients receiving cataract surgery, but the protective effect ceased to exist after three months. The structural protection of topical NSAIDs, however, can last for at least three months. Meanwhile, neither anti-VEGFs nor NSAIDs provided significant visual improvement. (4) Conclusions: Our study suggested that topical NSAIDs eye drops is an effective prevention strategy for macular edema after cataract surgery in diabetic patients.

## 1. Introduction

Macular edema that develop after cataract surgery in diabetic patients, including pseudophakic cystoid macular edema (PCME) and diabetic macular edema (DME), is one of the most common vision-threatening complications after uncomplicated cataract surgery in patients with diabetes [1]. Although PCME and DME have different underlying pathological mechanism [2], they have many similarities in morphological appearance. Marion et al. proposed an algorithm to differentiate between PCME and DME by spectral-domain optical coherence tomography (SD-OCT) images, but the authors also indicated its difficulty to deduce a gold standard method for absolute differentiation [3].

The incidence of PCME without risk factors after uncomplicated cataract surgery ranges between 0.1% and 2.3%, and the peaks are at approximately 5 weeks in a healthy population. However, the incidence of PCME is significantly increased up to 16.3% in patients with previous DME or diabetic retinopathy, whose blood–retinal barrier has already been compromised before surgery [4]. These hypotheses explained not only their similarities in OCT features, but their relationship between PCME and DME in patients with diabetes. Moreover, previous literature also showed that these two conditions could coexist [5,6]. It also raises the level of difficulty for differentiation. In our study, we use the term “macular edema after cataract surgery” to describe the union of these two disease entities.

Much effort was put to preventing macular edema after cataract surgery. Steroids and nonsteroidal anti-inflammatory agents (NSAIDs) were explored as prophylactic drugs in the form of eyedrops or intra-vitreal injection after cataract surgery [7,8,9]. Previous studies revealed that despite the significantly protective effect against the incidence of pseudophakic macular edema, there was no significant difference in visual improvement between patients receiving topical steroid, NSAIDs eye drops, and those receiving placebo eye drops [9]. Up to date, no consensus has been reached regarding the optimal strategy of preventing macular edema after cataract surgery after uncomplicated cataract surgery.

Additionally, previous literature revealed conflicting results in terms of the effectiveness of intravitreal injections of anti-VEGF to prevent macular edema after cataract surgery in diabetic patients without pre-existing macular edema [9,10]. Furthermore, there is a paucity of studies to compare the effectiveness of intravitreal anti-VEGF injection and topical NSAIDs eye drops as prophylactic strategies for macular edema after cataract surgery after uncomplicated cataract surgery in patients with diabetes [11,12].

The aim of the present study was to compare the effectiveness of intravitreal anti-VEGF and topical NSAIDs eye drops. Due to the possibility of co-existence of PCME and DME and the difficulty to differentiate these two disease entities, we consider the occurrence of both diseases as a single outcome of macular edema after cataract surgery. Furthermore, we applied network meta-analysis by organizing previous literature to infer the results that the ongoing debate about the optimal strategy to prevent macular edema after cataract surgery in patients with diabetes after uncomplicated cataract surgeries.

## 2. Materials and Methods

We conducted this systematic review and network meta-analysis according to the PRISMA guidelines1 (we registered the study on Prospero CRD, ID: 207987). The study used published data which was exempted from institutional review board approval.

### 2.1. Eligibility Criteria

Studies were eligible if they conformed to randomized controlled trials (RCTs) and if they only included diabetic patients with diabetic retinopathy. We only included trials that reported occurrence rates of post-operative macular edema or changes in best corrected visual acuity (BCVA).

To eliminate the treatment effect of anti-VEGF on pre-existing diabetic macular edema, trials were excluded if they include patients with pre-existing macular edema before surgery. We included trials without limitation of duration of treatment. However, at least four weeks of follow up was required in each trial.

### 2.2. Search Strategy

We searched for potential references from the electronic database: Ovid MEDLINE, PUBMED, and EMBASE databases from the inception of the databases through 31 July 2021. The search strategy consisted of using several relevant search terms, namely, “Diabetic Retinopathy”, “Cataract Extraction”, “Bevacizumab”, “Ranibizumab”, “Aflibercept”, and “Non-Steroidal Anti-Inflammatory Agents”, along with appropriate Boolean algebra. The full search strategy is listed in the Supplementary Materials.

### 2.3. Study Selection

Two authors (C.-A. Hsu and Y.-B. Chou) examined the search results according to the inclusion criteria to filter out eligible articles. Initial filtering was conducted through the examination of titles and abstracts. Non-English reports were translated via proper measures and further evaluated for eligibility afterwards. The full texts of potentially eligible articles were obtained and further examined. Any uncertainties regarding a study’s inclusion were resolved by consulting a third author (S.-C. Chi).

### 2.4. Data Collection and Risk of Bias Assessment

For each included trial, two authors (C.-A. Hsu and S.-C. Chi) extracted the data independently, including study design, participant characteristics, outcomes, and risk of bias with a standardized form. Specifically, pre-operative and post-operative BCVA or the change of BCVA before and after operations of included trials were extracted. For visual acuity in non LogMAR format, the differences in the numbers in ETDRS letters were transformed into logMAR differences before synthesizing the results [13]. The numbers of patients that developed macular edema after the operation in each trial were also extracted.

Two authors (C.-A. Hsu and Y.-B. Chou) performed the assessment of trial quality independently using the Risk of Bias 2 (RoB2) tool provided by Cochrane Collaboration for randomized controlled trials. Discrepancies regarding the grading of trials was resolved by consulting a third author (S.-C. Chi).

### 2.5. Data Synthesis and Analysis

#### 2.5.1. Qualitative Analysis

Trial design and participant characteristics were evaluated to determine heterogeneity among studies. Moreover, risk of bias of included trials were evaluated and synthesized in Supplementary Materials.

#### 2.5.2. Quantitative Analysis

Pairwise meta-analysis of both treatment strategies, intravitreal Anti-Vascular endothelial growth factor and topical Non-Steroidal Anti-Inflammatory Drug, were conducted. We used R version 4.0.3 (R Foundation for Statistical Computing, Vienna, Austria) to conduct the pairwise meta-analysis. The “meta” and “metafor” package were utilized to conduct the pairwise analysis. Risk ratio and mean difference was summarized for binary and continuous outcomes, respectively. Random-effects model was used with Sidik–Jonkman estimator. Frequentist models were then fitted utilizing R with “netmeta” package. Surface Under the Cumulative Ranking (SUCRA) scores were then calculated.

We utilized Funnels plots and egger’s test using R with “metafor” package to examine publication bias. “dmetar” package in R was used to conduct sensitivity analysis, including outlier identification and leave one out analysis. STATA 16 (StataCorp LP, College Station, TX, USA) was used to generate net graph. Node heat map and node splitting were also generated with “metafor” package in R.

## 3. Results

### 3.1. Study Inclusion and Demographics

**Figure 1:**
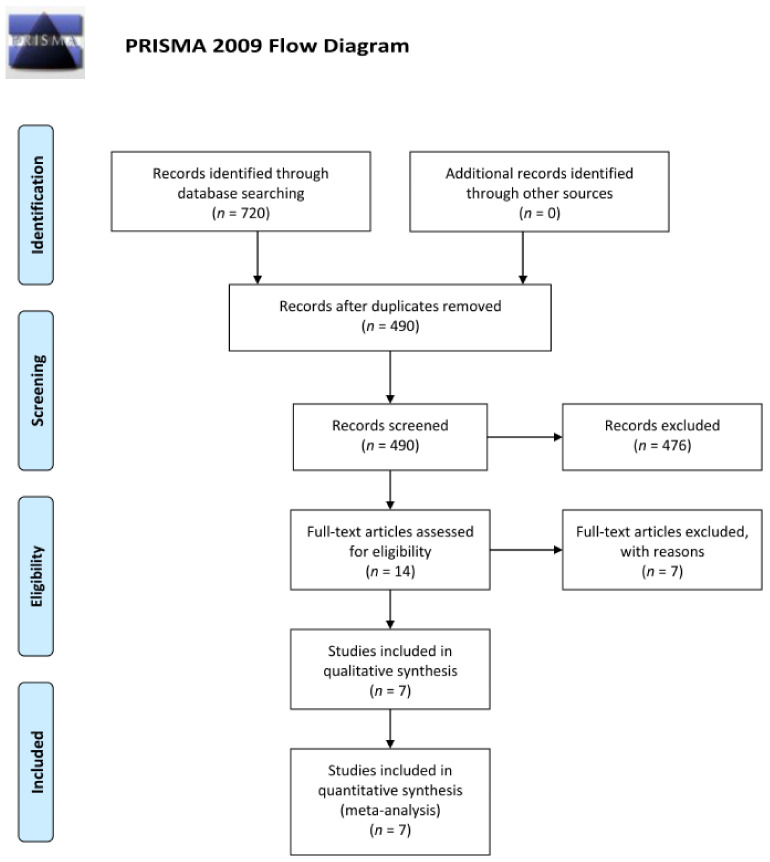
Flow diagram (from Moher et al., 2009) illustrating the steps of the systemic search of the past literature according to the PRISMA guidelines. For more information, visit www.prisma-statement.org (accessed on 9 December 2021).

Seven hundred and twenty records were subjected to title and abstract screening. After excluding duplicates, 490 records were eligible for title and abstract screening. Of these 490 records, 476 records were excluded during title and abstract screening. Full text of fourteen records were obtained [14,15,16,17,18,19,20,21,22,23,24,25,26,27]. In the 14 trials obtained, 7 trials were excluded due to inclusion of patient with pre-existing diabetic edema or absence of report of best corrected visual acuity and occurrence rate of post-operative macular edema. Eventually, 7 trials were included in the quantitative analysis [14,16,17,19,22,25,27] (Figure 1).

A total of 120 eyes received intravitreal injection of anti-VEGF, 719 eyes received topical non-steroidal anti-inflammatory drugs eye drops, and 762 eyes received sham injections or vehicle eye drops (Table 1). Of the included trials regarding intravitreal injections of anti-VEGF, all participants received the injection during cataract surgery. On the other hand, all participants from the topical NSAIDs eye drops trials received one month of NSAIDs eye drops after operation.

Among the included studies, the age of the intervention group ranged from 60 to 69 years, while that of the control group ranged from 62 to 68.1 years. The sex ratio of the studies ranged from 0.29 to 1.76 male-to-female in the intervention group and 0.50 to 1.38 male-to-female in the control group. Moreover, after transforming to the LogMAR format, the visual acuity of the intervention group ranged from 0.298 to 0.76, while that of the control group range from 0.316 to 0.72.

### 3.2. Quality Assessment

All the included eyes (*n* = 1601) came for randomized controlled trials. The RoB 2 tool (a revised Cochrane risk of bias tool for randomized trials) was used for quality assessment of the included trials, and the results are reported in the Supplementary Materials. Most of the trials received the judgement of “Some concern”. The main domain that caused concern was that of “Randomization”. While most of the trials involved some elements of randomization, only one trial reported the detailed information of the randomizing process.

### 3.3. Risk Ratio of Post-Operative Macular Edema

**Figure 2:**
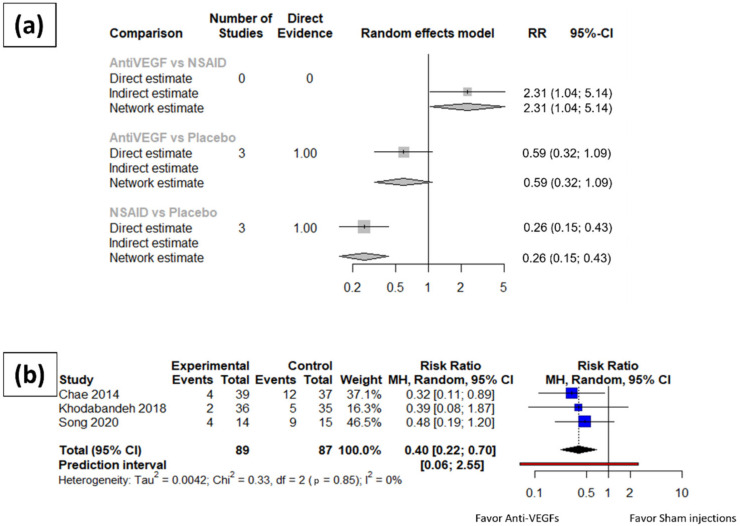
Meta-analysis results of the structural outcomes for anti-VEGFs and topical NSAID eye drops compared to placebo. (**a**) Network meta-analysis results of the incidence rate of macular edema three months after surgery. (**b**) Incidence rate of macular edema one month after surgery in anti-VEGF arms.

The risk ratio of post-operative macular edema at one month and three months after cataract surgery were synthesized. The synthesized results (Figure 2) revealed a significantly lower incidence rate of macular edema at three months after cataract surgery among the patients receiving topical NSAIDs eye drops comparing to those receiving placebo or vehicle eye drops (RR: 0.26, 95% CI: 0.15~0.43). On the other hand, no significant difference was detected in the incidence rate of macular edema at three months after cataract surgery among patients receiving intravitreal anti-VEGF injections and patients receiving sham injection (RR: 0.59, 95% CI: 0.32~1.09). Moreover, patients receiving intravitreal anti-VEGF injections had a significantly higher incidence rate of macular edema compared with patients receiving topical NSAIDs eye drops (RR: 2.31, 95% CI: 1.04~5.14).

Patients receiving anti-VEGF injections had a significantly lower incidence rate of macular edema one month after cataract surgery compared to those receiving placebo treatment (RR: 0.40, 95% CI: 0.22~0.70). Since only one trial of NSAIDs eye drops reported data eligible for quantitative analysis at one month after cataract surgery, the pairwise meta-analysis was omitted, and no network meta-analysis is available.

No asymmetry was found in either the three-month or one-month analyses. Moderate heterogeneity (I square equal to 58.2%, 95% CI: 0.0–88.1%) was found within the group of NSAID trials at three months after cataract surgery, while no significant heterogeneity (I square equal to 0.0%, 95% CI: 0.0–73.2% at three-month; I square equal to 0.0%, 95% CI: 0.0–37.6% at one month) was revealed in the anti-VEGF trials at either three months or one month after cataract surgery. Baujat diagnostics was further applied to the three-month meta-analysis results of the NSAID patients to reveal the contributor of the heterogeneity and reported in the Supplementary Materials.

### 3.4. Change of Best Corrected Visual Acuity (BCVA) after Cataract Surgery

**Figure 3:**
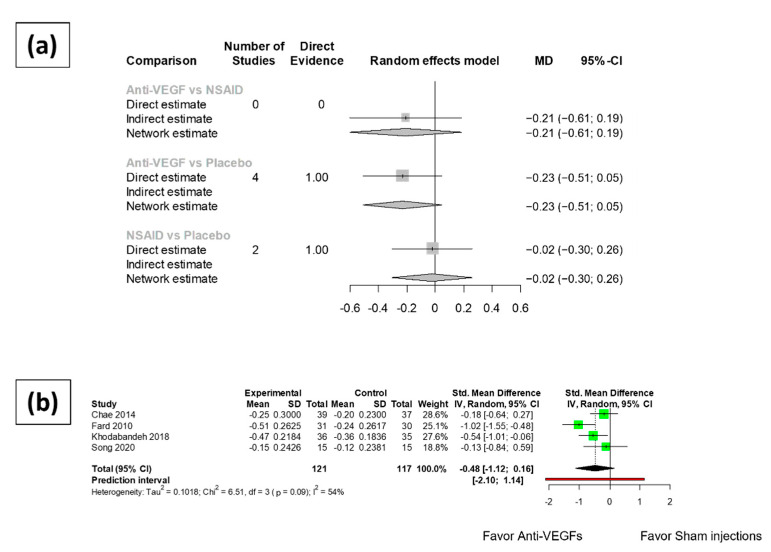
Meta-analysis results of the functional outcomes of anti-VEGFs and topical NSAIDs eye drops compared to placebo. (**a**) Network meta-analysis results of best corrected visual acuity (logMAR) at 3 months after surgery. (**b**) Best corrected visual acuity of anti-VEGF arms at 1 month after cataract surgery.

The mean difference of best corrected visual acuity change compared with pre-operative baseline in LogMAR format were compiled and summarized. The synthesized results (Figure 3) revealed no significant difference between the BCVA change of patients receiving intravitreal anti-VEGF injections at three months after cataract surgery and that of patients receiving sham injections (MD: −0.23, 95% CI: −0.51~0.05). There was also no difference detected in the BCVA change at three months after cataract surgery between the patients receiving NSAIDs eye drops and those receiving placebo or vehicle eye drops (MD: −0.02, 95% CI: −0.30~0.26). No significant difference was noted between the BCVA change at three months after cataract surgery in the patients receiving intravitreal anti-VEGFs and those receiving NSAIDs eye drops (MD: −0.21, 95% CI: −0.61~-0.19).

One month after cataract surgery, no significant difference was revealed in the BCVA change of patients receiving anti-VEGF injections and that of patients receiving sham injections (MD: −0.48, 95% CI: −1.12~0.16). Only one NSAIDs eye drops trial reported data eligible for meta-analysis at one month after cataract surgery. Therefore, the meta-analysis of functional outcomes at one month after cataract surgery for the NSAIDs eye drops trials was omitted.

Asymmetry was found in the one-month analysis of the anti-VEGF group. The study by Fard et al. reported significantly better visual acuity compared to the placebo group. However, the result of Egger’s test was not significant. High heterogeneity was detected (75.4%, 95% CI: 0.0–94.4%) among the NSAID arms. Moderate heterogeneity (I square equal to 54.7%, 95% CI: 0.0–83.3%) was found within the group of anti-VEGF arms at one month after surgery, while no significant heterogeneity (I square equal to 0.0%, 95% CI: 0.0–25.6%) was revealed at three months after surgery.

## 4. Discussion

In our literature review, topical steroid was considered as the standard of care after cataract surgery in participating clinical trials, but it is yet inconclusive to determine the efficacy between NSAIDs eye drops and intravitreal anti-VEGF injection in preventing macular edema after cataract surgery after cataract surgery in diabetic patients. Therefore, we aim to apply network meta-analysis to quantitatively compare these two strategies.

In the present study, the risk ratio (RR: 2.31, 95% CI: 1.04~5.14) for macular edema at third month after cataract surgery was significantly higher in patients receiving anti-VEGF injections compared to those receiving NSAIDs eye drops. This result confirmed the superiority of NSAIDs eye drops in preventing structural changes of the macula [9]. Interestingly, the preservatives in NSAIDs eye drops were correlated with the incidence of macular edema after cataract surgery [28]. Some prospective studies attempted to explore the role of preservative-free NSAIDs eye drops after cataract surgery [29,30], which implied that the preservative-free NSAIDs may provide stronger protective effect in macular edema after cataract surgery after cataract surgery.

In addition, previous studies demonstrated ineffectiveness of intravitreal anti-VEGF injections in structural protection at third months after cataract surgery [11,31]. Our study revealed that intravitreal anti-VEGF injections may provide short-term protective effect in structural change of the macula. The concentrations of anti-VEGF at one month after injection is less than one percent compared to that of one day after injection in a rabbit model [32]. Therefore, duration of this short-term protective effect may be related to the pharmacokinetic properties of anti-VEGF in vitreous space.

In previous studies, there was still argument over the improvement of BCVA in topical NSAIDs or intravitreal anti-VEGF injections [7,9,10]. Laursen et al. reported the lack of significantly visual improvement in the NSAIDs group compared to the placebo group. In contrast, Singh et al. conducted two of the largest NSAID trials and reported a significant visual improvement in the NSAIDs group compared to the control group. The limited number of patients in clinical trials and the diversity of visual acuity testing constrained the application of quantitative synthesis in previous studies. In the present study, after applying proper transformation of ETDRS to LogMAR format and including newly published trial [20], there was no significant visual improvement in the patients who received NSAIDs eye drops or intravitreal anti-VEGF injections at third month after cataract surgery. These results implied that the difference in structural protection was inconsistent with its effect on visual acuity, but other visual function outcomes, including contrast sensitivity and quality of life, should be explored in future study.

The type of anti-VEGF is a crucial factor for treatment efficacy in macular edema. A previous network meta-analysis reported that intravitreal injection of aflibercept showed better functional outcomes than either ranibizumab or bevacizumab, whereas ranibizumab showed greater improvement in central retinal thickness [10]. However, no final conclusion has yet been reached on this matter. Longitudinal studies should be conducted in the future to compare the efficacy of different anti-VEGFs in preventing macular edema after cataract surgery.

There are several limitations in the present study. The primary limitation is the lack of direct evidence for the comparison of anti-VEGFs and NSAIDs. This is common in network meta-analysis due to the comparison could only come from indirect evidence. Although it might impinge on the validity of the analysis, it also highlighted the critical comparison between topical NSAIDs and intravitreal anti-VEGS injections in clinical prevention of macular edema after cataract surgery in diabetic patients. Since no prior evidence could be obtained from the current literature, our study represents the first study to report such results.

Secondly, while the ETDRS letters were transformed to LogMAR scores according to previous studies, there might be some inevitable error in the transformation since the transformation was based on some assumptions regarding the distributions of visual acuity scores in the population that might not be applicable to all the enrolled studies. Accordingly, the meta-analysis of the BCVA data might not be completely accurate.

Thirdly, the clinical trials included in our meta-analysis presented unavoidable heterogeneity in terms of population, treatment protocol, and trial design. Two of the trials [17,22] included patients without diabetic retinopathy. The study performed by Entezari et al. [22] was not included in the final quantitative analysis of the incidence of macular edema since they did not report this data. On the other hand, Khodabandeh et al.’s study [17] also enrolled patients without diabetic retinopathy. However, further sensitivity analysis revealed no apparent contribution of heterogeneity from this study. Although we tried to explore other sources of heterogeneity, it is difficult to perform meta-regression on additional factors, such as types of anti-VEGFs or NSAIDs eyedrops, due to insufficient number of clinical trials.

Despite above limitations, the results in the present study provide evidence to support the difference between topical NSAIDs and intravitreal anti-VEGS injections in clinical prevention of macular edema after cataract surgery in diabetic patients.

## 5. Conclusions

In conclusion, our study suggests that topical NSAIDs eye drops are likely better than anti-VEGFs in primary prevention of macular edema after cataract surgery in diabetic patients undergoing cataract surgery. Anti-VEGFs only demonstrated short-term structural benefit at one month after cataract surgery. Although the difference in structural protection was inconsistent with its effect on visual acuity, topical NSAIDs may provide longer benefit in other patient-reported visual function outcomes.

## Figures and Tables

**Table 1:** Basic demographic data of selected studies.

	Intervention	Sample Size (Intervention/ Control)	Age of Intervention Group, Average (SD)	Age of Control Group, Average (SD)	Sex of Intervention Group (M/F, Sex Ratio)	Sex of Control Group (M/F, Sex|Ratio)	Severity of Diabetic Retinopathy in Intervention Group No/Mild/Moderate/Severe/PDR	Severity of Diabetic Retinopathy in Control Group No/Mild/Moderate/Severe/PDR	Initial BCVA of Intervention Group	Initial BCVA of Control Group
	Anti-VEGF arms									
Fard 2010	Bevacizumab	63 (31/32)	62 (5)	60 (4)	15/15, 1.00	18/13, 1.38	Not reported (Moderate to Severe NPDR cases included)	Not reported (Moderate to Severe NPDR cases included)	0.75 (0.17)	0.72 (0.18)
Khodabandeh 2018	Bevacizumab	69 (35/33)	61.7 (6.4)	66.3 (10.8)	8/28, 0.29	13/22, 0.59	24/12/0/0/0	18/17/0/0/0	0.54 (0.21)	0.46 (0.16)
Chae 2014	Ranibizumab	76 (39/37)	62.9 (14.2)	67.2 (8.3)	21/18, 1.17	20/17,1.18	Not reported (NPDR or stable PDR after PRP cases included)	Not reported (NPDR or stable PDR after PRP cases included)	0.50 (0.25)	0.52 (0.25)
Song 2020	Aflibercept	30 (15/15)	66 (Not reported)	66 (Not reported)	9/6, 1.50	5/10, 0.50	0/5/4/1/5	0/5/5/1/4	0.298 (0.612) ^1^	0.316 (0.85) ^1^
	NSAID arms									
Entezari 2016	Diclofenac	108 (54/54)	67 (8)	69 (6)	21/33, 0.64	27/27, 1.00	41/34/22/11/0	21/15/13/5/0	0.96 (0.36)	1.1 (0.28)
Pollack 2016	Nepafenac	175 (87/88)	68.1 (8.6)	69.4 (7.6)	51/29, 1.76	44/36, 1.22	0/58/21/1/0	0/27/22/1/0	0.434 (0.256) ^1^	0.396 (0.242) ^1^
Singh 2017 A	Nepafenac	589 (289/300)	66.8 (8.5)	66.8 (8.3)	131/158, 2.26	134/166, 0.81	0/40/255/3/0	0/44/253/3/0	0.46 (0.242) ^1^	0.44 (0.22) ^1^
Singh 2017 B	Nepafenac	582 (289/293)	67.7 (8.5)	68.1 (8.4)	140/149, 0.94	144/149, 0.97	0/29/260/0/0	0/33/257/3/0	0.508 (0.28) ^1^	0.504 (0.248) ^1^

^1^ Converted from ETDRS letters; Nonproliferative diabetic retinopathy (NPDR); Proliferative diabetic retinopathy (PDR).

## Data Availability

No new data were created or analyzed in this study. Data sharing is not applicable to this article.

## Supplementary Materials

The following are available online at https://github.com/nfwya/Macular-edema-after-cataract-surgery-DM-Meta-analysis (accessed on 25th February 2022).
